# A local-scale One Health genomic surveillance of *Clostridioides difficile* demonstrates highly related strains from humans, canines, and the environment

**DOI:** 10.1099/mgen.0.001046

**Published:** 2023-06-22

**Authors:** Charles H. D. Williamson, Chandler C. Roe, Joel Terriquez, Heidie Hornstra, Samantha Lucero, Amalee E. Nunnally, Adam J. Vazquez, Jacob Vinocur, Carmel Plude, Linus Nienstadt, Nathan E. Stone, Kimberly R. Celona, David M. Wagner, Paul Keim, Jason W. Sahl

**Affiliations:** ^1^​ The Pathogen and Microbiome Institute, Northern Arizona University, Flagstaff, AZ, USA; ^2^​ Flagstaff Medical Center, Flagstaff, AZ, USA

**Keywords:** *Clostridioides difficile*infection, comparative genomics, One Health

## Abstract

Although infections caused by *Clostridioides difficile* have historically been attributed to hospital acquisition, growing evidence supports the role of community acquisition in *C. difficile* infection (CDI). Symptoms of CDI can range from mild, self-resolving diarrhoea to toxic megacolon, pseudomembranous colitis, and death. In this study, we sampled *C. difficile* from clinical, environmental, and canine reservoirs in Flagstaff, Arizona, USA, to understand the distribution and transmission of the pathogen in a One Health framework; Flagstaff is a medium-sized, geographically isolated city with a single hospital system, making it an ideal site to characterize genomic overlap between sequenced *C. difficile* isolates across reservoirs. An analysis of 562 genomes from Flagstaff isolates identified 65 sequence types (STs), with eight STs being found across all three reservoirs and another nine found across two reservoirs. A screen of toxin genes in the pathogenicity locus identified nine STs where all isolates lost the toxin genes needed for CDI manifestation (*tcdB*, *tcdA*), demonstrating the widespread distribution of non-toxigenic *C. difficile* (NTCD) isolates in all three reservoirs; 15 NTCD genomes were sequenced from symptomatic, clinical samples, including two from mixed infections that contained both *tcdB+* and *tcdB*- isolates. A comparative single nucleotide polymorphism (SNP) analysis of clinically derived isolates identified 78 genomes falling within clusters separated by ≤2 SNPs, indicating that ~19 % of clinical isolates are associated with potential healthcare-associated transmission clusters; only symptomatic cases were sampled in this study, and we did not sample asymptomatic transmission. Using this same SNP threshold, we identified genomic overlap between canine and soil isolates, as well as putative transmission between environmental and human reservoirs. The core genome of isolates sequenced in this study plus a representative set of public *C. difficile* genomes (*n*=136), was 2690 coding region sequences, which constitutes ~70 % of an individual *C. difficile* genome; this number is significantly higher than has been published in some other studies, suggesting that genome data quality is important in understanding the minimal number of genes needed by *C. difficile *. This study demonstrates the close genomic overlap among isolates sampled across reservoirs, which was facilitated by maximizing the genomic search space used for comprehensive identification of potential transmission events. Understanding the distribution of toxigenic and non-toxigenic *C. difficile* across reservoirs has implications for surveillance sampling strategies, characterizing routes of infections, and implementing mitigation measures to limit human infection.

## Data Summary

Sequencing data has been deposited at the National Centre for Biotechnology and Information (NCBI) under BioProjects PRJNA728705, PRJNA309189 and PRJNA438482. Individual accession numbers are included in Table S1. Publicly available software and data are described within the article.

**Impact Statement**

*
Clostridioides difficile* infection (CDI) can include symptoms ranging from asymptomatic carriage to severe disease and death. In this study, we analysed whole genome sequence data from 562 *C *. *difficile* isolates collected from three sources (clinical, environmental and dog) to understand the distribution of this species within Flagstaff, AZ, USA. Diverse isolates (65 different multi-locus sequence types) were identified in Flagstaff, and *C. difficile* was widespread in the environment (~35 % of soil and water samples yielded isolates). Comparative genomic analyses were used to identify very closely related isolates that could indicate transmission events. Approximately 19 % of clinical isolates were associated with potential healthcare-associated transmission (though asymptomatic transmission was not considered), and we identified putative transmission between canine and soil reservoirs and between the environment and humans. Our study examines *C. difficile* within a One Health framework and could improve surveillance and mitigation of CDI.

## Introduction

In 1978, multiple scientific studies identified *Clostridioides difficile* as the causal agent of antibiotic-associated pseudomembranous colitis [1–4]. *C. difficile* causes disease in humans largely through the action of two toxins encoded by *tcdA* (Toxin A) and *tcdB* (Toxin B) [5], located on the pathogenicity locus (PaLoc) [6]. The PaLoc is 19 kb region of the genome that also includes a RNA polymerase sigma factor (*tcdR*) [7], a negative regulator (*tcdC*) [8], and a gene associated with holin activity (*tcdE*) [9]. In addition to the PaLoc, the binary toxin (CDT) has been described in *C. difficile *, although its role in virulence has not been fully elucidated [10]. *C. difficile* infection (CDI) can result in a wide range of outcomes, ranging from asymptomatic carriage to severe forms of disease resulting in death. CDI was long considered a predominately hospital-acquired infection [11, 12], and much attention has been given to monitoring and limiting transmission of *C. difficile* within healthcare settings (e.g. antibiotic stewardship) [13–15]. However, reports have described an increase in the global incidence of community-acquired *C. difficile* infections (CA-CDI) that impact populations thought to be at lower risk of infection [16–19]. As *C. difficile* has been identified in raw meat products, companion animals, asymptomatic humans, and throughout the environment, recent research has investigated *C. difficile* and CDI within a One Health framework [17].

*C. difficile* has been isolated from the gut of most mammals [20] including cattle, horses, pigs, and poultry [21–27] as well as companion animals such as dogs and cats [28–30]. Studies have also demonstrated the prevalence of *C. difficile* in the environment, including sand playgrounds [31], public gardens [32], rivers [33], soil [17, 34, 35], water [36], and environments in contact with farm animals [23]. This apparently near ubiquitous presence is likely due to the pathogen’s ability to form oxygen resistant spores [34] and persist in a dormant state until a new mammalian host is found [37]. The potential transmission of *C. difficile* between animals and humans has been investigated [38–42], though direct transmission of *C. difficile* between animals and humans has only been rarely reported [43, 44]. Although researchers have described *C. difficile* as omnipresent in humans, non-human animals, and the environment, a causal connection between environmental *C. difficile* isolates and human or animal disease is difficult to definitively demonstrate [17, 41].

Whole genome sequencing and comparative genomics have allowed for more comprehensive and higher resolution analyses of *C. difficile* isolates than older, sub-genomic techniques. Much of the original research on genotyping *C. difficile* focused on 16–23S ribotyping [45], a relatively low resolution fingerprinting technique [46]. Many of the important *C. difficile* groups, including the ‘hypervirulent’ ST1/RT027 [47] and ST11/RT078 [48] groups, have also been identified through multi-locus sequence typing (MLST) [49]. These techniques are being replaced by whole genome sequencing and comparative genomics that have allowed researchers the ability to track transmission events [13], understand population diversity [50, 51], and characterize the pan-genome of the pathogen [52], including genes associated with antimicrobial resistance [53] and differential virulence [54].

There is a current need to explore the human disease burden associated with *C. difficile* from companion animals and the environment. Incorporating *C. difficile* isolates from these diverse settings along with clinical isolates within a phylogenetic framework could allow for accurate identification of host origin as well as inference of directionality of transmission events. Here we have applied an optimal approach of high-resolution genomic analyses within a One Health framework to improve our understanding of the role that *C. difficile* plays in humans, animals, and the environment in Flagstaff, Arizona, USA. Our study includes 401 clinical, 50 canine, and 111 environmental isolates collected over the course of 5 years. Flagstaff is a geographically isolated city with a single hospital system allowing for comprehensive sampling of clinical *C. difficile* infections, which then allows for characterizing genomic overlap between sequenced *C. difficile* isolates across reservoirs. These isolates were compared using multiple high-resolution genomic analyses to identify overlap between *C. difficile* causing human disease with isolates found in companion animals and the environment.

## Methods

### Sample collection and *C. difficile* isolation

**Fig. 1.**
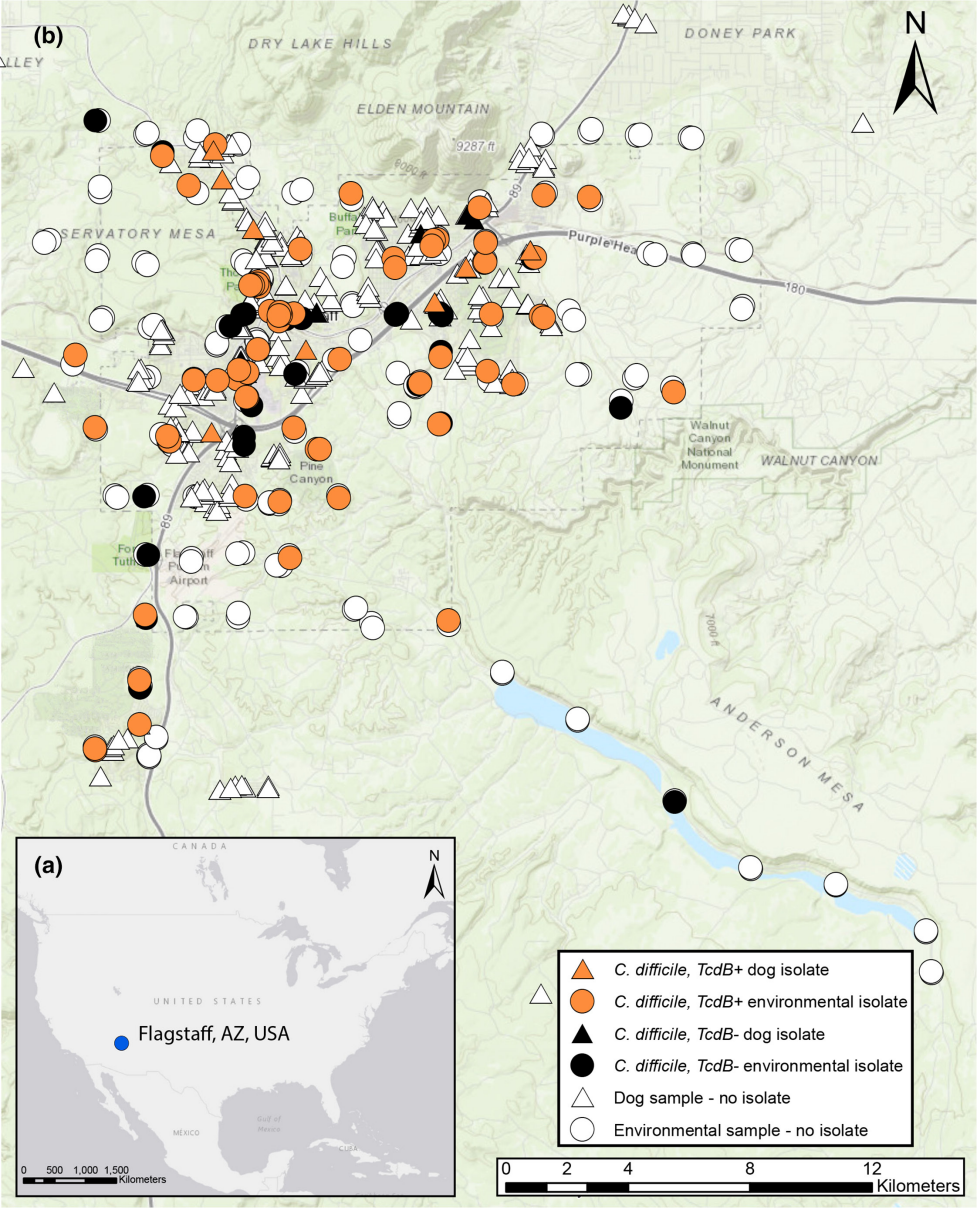
Map of sample collection sites in Flagstaff, Arizona, USA. (**a**) The blue dot marks the location of Flagstaff, Arizona, USA. (**b**) Triangles indicate the locations of collected dog faecal samples, and circles indicate the collection sites for environmental samples (soil and water). The colour of these shapes describes if the samples were positive for *C. difficile* (isolate identified as *C. difficile* by whole genome sequencing) and if the isolate contained the *tcdB* toxin gene (see key). This map was created using ArcGIS software by Esri.

*Environmental sample collection and processing*. Soil (*n*=210) and water (*n*=50) samples were systematically collected around Flagstaff, Arizona, USA in 2017 and 2019 to determine the prevalence of *C. difficile *. The sampling effort and isolation of *Clostridioides* from environmental samples have been described previously [55]; samples from two additional sites were collected to optimize protocols for environmental enrichment and isolation of *C. difficile* (ES-S-ARDP, ES-S-ARDRB) and processed as described previously [55]. A map of sampling sites around Flagstaff is included in Fig. 1. Briefly, samples were enriched in C diff Banana Broth (Hardy diagnostics) at 36–37 °C for 72 h; enrichment culture was plated onto taurocholate-cefoxitin-cycloserine-fructose agar (TCCFA) and incubated anaerobically for 24 h at 36 °C; then, samples were streaked on brain heart infusion agar supplemented with 0.03% l-cysteine (BHIS) and incubated anaerobically for 24 h at 36 °C (for more detailed methods see [55]). From each enriched and plated sample, up to ten individual colonies were screened with a qPCR assay targeting *tcdB* [28] and with an assay targeting a biomarker thought at the time to be specific to *C. difficile* [28, 55]. For some samples, multiple isolates were putatively identified as *C. difficile *. For these samples multiple isolates were selected for sequencing based on MLST results as well as a qPCR assay targeting *tcdB* [28]; isolates with different MLST or toxin profiles were sequenced. Isolates putatively identified as *C. difficile* were then sequenced as described below and species composition was confirmed through average nucleotide identity (ANI) analyses using PYANI v0.2.11 [56]; an ANI threshold of >95 % was used to confirm species membership [57].

*Companion animal samples*. Canine faecal samples were collected around Flagstaff in 2014 and 2015, which has been described previously [28]; 36 *C *. *difficile* genomes from that sampling effort are included in this study (Table S1). Additional canine faecal samples were collected opportunistically from the environment (*n*=200) or donated by third parties (*n*=136) for surveillance efforts in Flagstaff between 2017 and 2019; putative *C. difficile* isolates from enrichments were whole genome sequenced (*n*=14) and included in the current study.

*Clinical samples. C. difficile* positive stool samples*,* based on the Cepheid Xpert *C. difficile* assay (Cepheid, Sunnyvale, CA), were collected from two northern Arizona healthcare facilities between 2016 and 2019 under IRB protocol No. 764034-NAH and were stored at −80 °C until processing; all specimens were deidentified before transfer to NAU. The processing of clinical stool samples in the laboratory was performed using protocols published previously [58]. For each sample, ten colonies were separately processed for MLST and *tcdB* presence. Multiple colonies were sequenced when more than one sequence type (ST) was identified in a single sample.

### DNA sequencing and genome assembly

Sequencing libraries were generated for dual-indexed, paired-end sequencing on Illumina platforms using previously described methods [28]. All genomes were assembled using SPAdes v3.13.0 [59] and polished with Pilon 1.24 [60]. Depth of coverage was calculated across each contig by aligning reads with minimap2 v2.17 [61] and counting per base coverage with Samtools v1.2 [62]. The identity of contigs was performed by aligning 200 nucleotides from each against the GenBank [63] nucleotide database with blastn v2.11.0 [64]. Contigs that either contained an anomalously low depth of coverage or aligned against known contaminants were manually removed. Raw reads and assemblies were deposited in NCBI under BioProject PRJNA728705. Information for Flagstaff genomes is compiled in Table S1. Genomic data for Flagstaff isolates that were sequenced and analysed previously (*n*=66) [28, 58, 65] were also included in this study (Table S1; BioProjects PRJNA309189, PRJNA438482).

### External genome assembly download and curation

*C. difficile* genome assemblies were downloaded from the GenBank assembly database on 27 September 2022 using the ncbi-genome-download tool (https://github.com/kblin/ncbi-genome-download), resulting in 14 113 genome assemblies. Assemblies were removed from the dataset if all seven MLST loci were not identified in the assembly (see below for MLST method, 70 assemblies failed criteria), if the assembly had greater than 500 contigs (509 assemblies failed criteria), if the assembly had an anomalous size (accepted range: 3638868–5023210, 14 assemblies failed criteria) or GC content (accepted range: 0.280–0.309, 12 assemblies failed criteria), or if the assembly was generated by a third party (81 assemblies failed criteria and removed to avoid duplicate assemblies) [66]; 13 848 assemblies remained after filtering. To generate a reference genome set, genomes were dereplicated with the genome-dereplicator tool (https://github.com/rrwick/Assembly-Dereplicator) at the default distance threshold, resulting in 148 genomes. These genome assemblies were compared with PYANI using the MUMmer v3.23 [67] alignment option, and assemblies that were outliers to *C. difficile* using an ANIm cutoff of 95 % [55, 68] were removed from the data set (*n*=12); these genomes grouped with previously-described cryptic *C. difficile* species [55]. The final reference set included 136 genome assemblies (Table S2).

### *In silico* MLST typing

Multi-locus sequencing typing of all genome assemblies was conducted with FastMLST v0.0.15 [69], which utilizes the typing scheme available from PubMLST [49].

### Core genome SNP analyses

Genome assemblies from Flagstaff genomes (*n*=562) and the reference set (*n*=136) were aligned against the completed reference genome, CD630 (GCA_000009205.2), using NUCmer v3.1 [67] and SNPs were called with NASP v1.2.1 [70]; SNPs identified in duplicated regions, based on a reference self-alignment with NUCmer, were filtered from all downstream analyses. A maximum-likelihood phylogeny was inferred on the concatenated SNP alignment with IQ-TREE v2.1.2 [71] using the integrated ModelFinder method [72]. Trees were visualized in iTOL [73].

To provide a high resolution SNP analysis within MLST groups of interest, Illumina sequencing reads were aligned to appropriate reference genomes with minimap2 (v2.24) and SNPs were identified with GATK4 (v4.1.8.1) [74]. Variant call format (VCF) files were processed within NASP v1.2.1 using a depth of coverage filter of 10× and a proportion filter of 0.9 [70]. The genomic search space for SNP analyses was determined by first filtering duplicated regions from the reference based on a reference self-alignment with NUCmer. All remaining positions in the reference genome that contained a valid nucleotide call (A,T,G,C) in at least one genome were considered to be part of the genomic search space for SNP comparisons. This reference-based genomic search space size describes the maximum number of positions considered for pairwise comparisons for genomes in each sequence type. Pairwise SNP distances were calculated with snp-dists (v0.8.2 - https://github.com/tseemann/snp-dists).

### Comparative genomic and pan-genomic analyses

Genome assemblies were annotated with Bakta v1.5.1 [75], and the resulting output files (‘.gff3’) were used to determine the core and pan-genome structure with Panaroo v1.2.3 [76]. To identify the conservation of coding region sequences (CDSs) between groups of interest, the Large-Scale Blast Score Ratio (LS-BSR) v1.2.3 [77] was used to screen Bakta-identified CDSs. To accomplish this, a reference bit score was generated by self-aligning coding regions using BLAT v36×2 [78], and these regions were then aligned against every genome in the sample set to calculate a query bit score. blast Score Ratios (BSRs) [79] were calculated by dividing the query bit score by the reference bit score. A BSR threshold of >0.80 was used to identify core CDSs within each group.

### *In silico* gene screen

A set of previously characterized toxin and regulatory genes were screened against genomes with blastn in conjunction with LS-BSR as described above. Genes screened included: *tcdA* (NC_013315.1 : 718474–726606) *tcdB* (NC_013315.1 : 710024–717124), *tcdC* (NC_013315.1 : 726941–727620), *tcdE* (NC_013315.1 : 717246–717746), *tcdR* (NC_013315.1 : 709139–709693), *cdtA* (NC_013315.1 : 2839368–2840759), *cdtB* (NC_013315.1 : 2840812–2843442), and *cdtR* (NC_013315.1 : 2838232–2838978). Toxin gene presence/absence within Flagstaff isolate genomes was also evaluated by aligning Illumina sequencing reads to reference sequences with minimap2 v2.24 [61] and calculating the breadth of coverage. Genes with a breadth of coverage greater than 75 % at a depth of coverage of 3× were considered as present.

We screened the peptide sequences of *tetM* (WP_110370588.1) and *ermB* (MBM9399818) against all genomes with TBLASTN/LS-BSR. TetM has been associated with resistance to tetracycline [80] and ErmB has been associated with resistance to erythromycin [81]. We also screened for an aminoglycoside phosphotransferase gene (aphA-3, AAA98050.1), an *erm* gene recently associated with erythromycin resistance in *C. difficile* [82], and coding regions from the pCD-METRO plasmid associated with metronidazole resistance [83].

### Pairwise SNP diversity within samples

To examine possible within-sample SNP diversity, eight colonies were separately extracted, sequenced, and analysed for each of five clinical samples (HS-FS-000222, HS-FS-000165, HS-FS-000148, HS-FS-000073, HS-FS-000190) and one dog sample (DGF-0048). In each case, colonies were randomly picked from a plate and were processed with the same DNA extraction/sequencing approaches described above. For each sample, all isolate sequence data were aligned back against an assembly from that same sample with NASP and SNPs were identified using the approaches described above.

### Sequence type 42 gene content analysis

CDSs were identified from the GenBank annotation of public genomes that were associated with previously identified ST42 genomic islands [84]. The nucleotide sequence from these CDSs (Table S3) were screened against all ST42 genomes (*n*=761) with LS-BSR/BLAT and CDSs with a BSR ≥0.9 were identified. We also identified the pan-genome of ST42 genomes with Bakta and Panaroo, mapped those regions back against genomes with BLAT/LS-BSR, and identified CDSs that were differentially conserved across phylogenetic clades within ST42.

## Results

### Diverse STs were found across all three sampled reservoirs

**Fig. 2.**
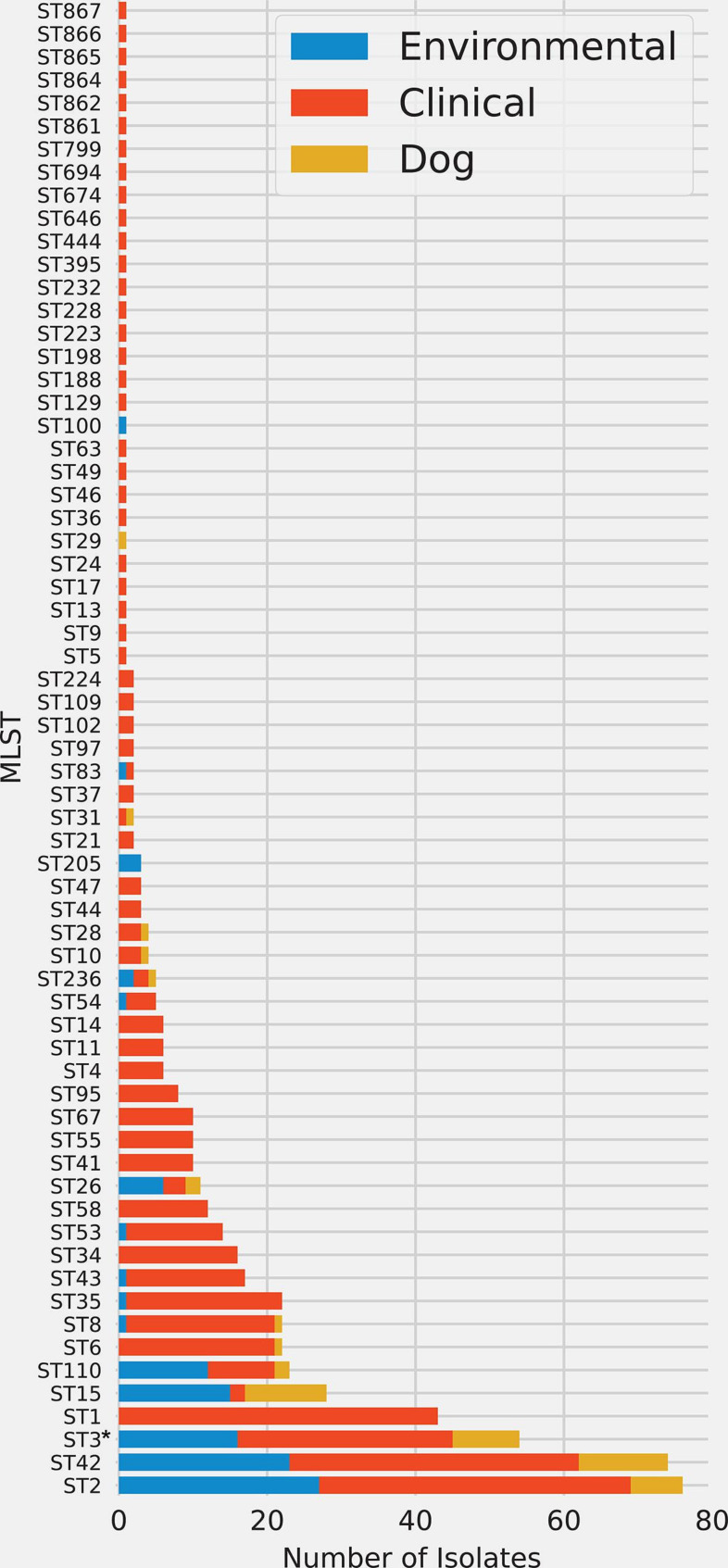
Bar chart indicating the counts of isolates collected for each sequence type (MLST). Colours indicate the reservoir (environmental, clinical or dog) from which isolates were sampled. ST3 includes both non-toxigenic (*n*=28) and toxigenic (*n*=26) isolates, which are present in two separate monophyletic clades in the core genome phylogeny (Fig. 3) and are described separately in this paper (see main text).

A total of 496 new isolates were sequenced in this study and were combined with a set of 66 previously published genomes from Flagstaff isolates (Table S1) to characterize the genomic diversity of *C. difficile* within a limited geographic area. Clinical isolates originated from a single healthcare system, whereas environmental and dog isolates originated from samples collected throughout the community (Fig. 1, samples collected from an area of ~625 km^2^). Based on an analysis of genomes sequenced for clinical (*n*=401), dog faeces (*n*=50), and environmental (*n*=111) isolates, we identified 65 unique STs (Fig. 2), with eight of them found across all three reservoirs and nine found across two reservoirs. Six novel STs were identified in this study, all from clinical samples, and include ST861, ST862 and ST864-ST867.

### The core genome phylogeny demonstrates the diversity of Flagstaff isolates

**Fig. 3.**
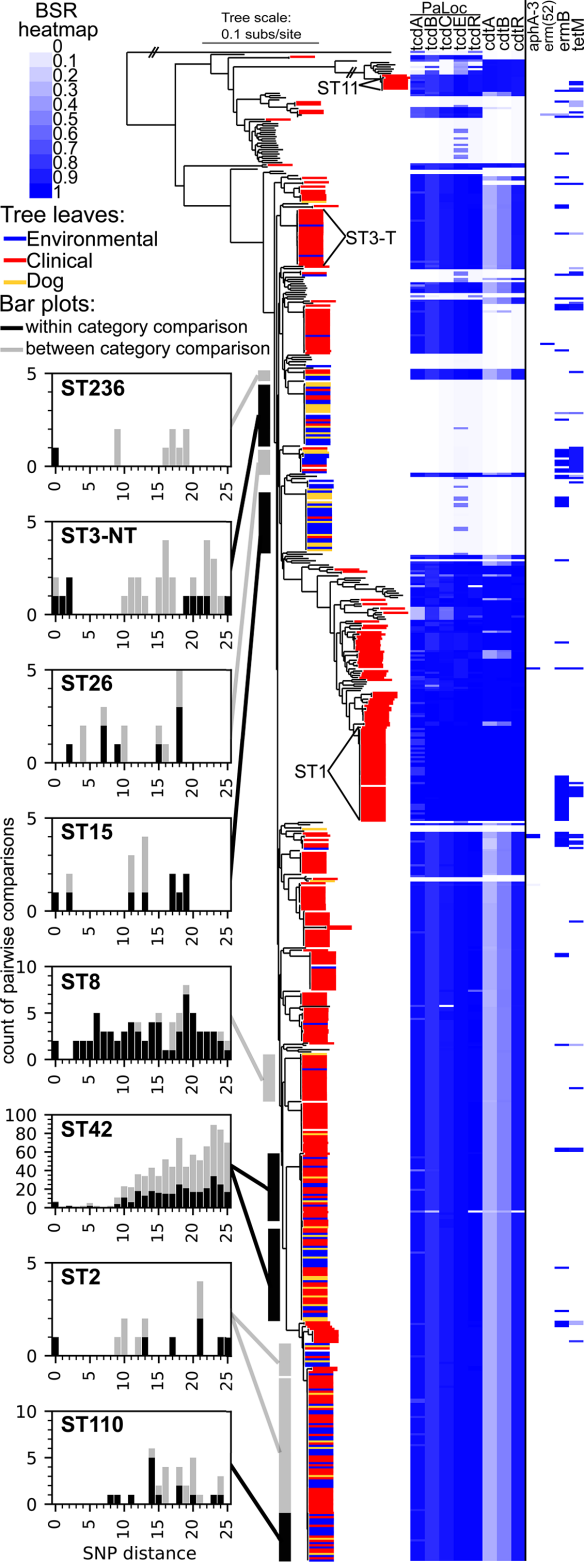
A core-genome SNP phylogeny is presented in the middle of the figure. Isolates from Flagstaff, AZ (this study) are highlighted: blue - environmental, red - clinical, yellow - dog. The heatmap on the right side of the figure displays information about the presence of genes of interest based on BSR values (see Methods and BSR heatmap key at top left corner of figure). Data for toxin genes and genes associated with antibiotic resistance are presented. Bar plots on the left side of the figure present the counts of isolate pairs that differ by 25 or fewer SNPs for sequence types that include all three reservoirs (environmental, clinical and dog). Black bars show counts for within category isolate pairs (e.g. two environmental isolates), and grey bars show counts for between category isolate pairs (e.g. one clinical isolate and one environmental isolate).

The core genome of the 698 *C *. *difficile* genomes processed in this study, based on NASP, consisted of 2.27M nucleotides, which constitutes ~53 % of the size of the CD630 chromosome (accession=NC_009089); this core genome space excludes repeat regions or any position that contains a missing or ambiguous nucleotide character. The core genome based on Panaroo, which focuses on coding region sequences, was 2690 CDSs; this is ~70 % of the total CDSs annotated in CD630 (*n*=3834). The core genome SNP phylogeny of Flagstaff *C. difficile* genomes and representative sequences (Fig. 3) demonstrates the broad diversity of *C. difficile* sequenced in Flagstaff over the course of this study, as reference genomes and genomes from Flagstaff are interspersed throughout the phylogeny.

### Toxin screen

The presence of genes in the pathogenicity locus (PaLoc) were largely conserved across clades (Fig. 3). However, multiple clades of non-toxigenic *C. difficile* were sequenced from Flagstaff, including clades containing isolates from all three sources (e.g. ST15), demonstrating that the loss of the PaLoc has occurred across independent lineages. For most *tcdB* ^+^ genomes, all genes in the PaLoc were identified (Fig. 3), although some genomes were *tcdA* ^-^/*tcdB* ^+^. Unsurprisingly, 386 of 401 clinical isolate genomes (>96 %) were *tcdB^+^*, as samples were pre-screened with a *tcdB* assay in the clinical laboratory. Two of the *tcdB* ^-^ isolates were obtained from clinical samples that also yielded a *tcdB* ^+^ isolate. For the remaining 13 isolates obtained from clinical samples, it is unclear if the clinical toxin assay returned a false positive, or if the toxigenic isolate could not be grown from stool samples in the laboratory.

Overall, *C. difficile* isolates were obtained and whole genome sequenced from ~9 % of canine faecal samples. Approximately 13 % of samples collected in 2014–2015 yielded isolates, whereas ~4 % of samples collected from 2017 to 2019 yielded isolates. An *in silico* screen of toxin genes against the canine *C. difficile* isolate genomes (*n*=50) identified 26 that were *tcdB* ^+^ (52 %). Some of these genomes represent multiple isolates obtained from a single faecal sample. For example, three isolates sequenced from sample DGF-0217 that were all associated with distinct STs (ST15, ST42, ST31); two of the three genomes (DGF-0217–01, DGF-0217–03) were *tcdB* ^-^, while one (DGF-0217–02) was *tcdB* ^+^.

*C. difficile* isolates were obtained and whole genome sequenced from 91 of 262 soil/water samples (~35 %) collected in Flagstaff, AZ, USA. An *in silico* tcdB gene screen against environmental genomes (*n*=111) identified 71 that were *tcdB* ^+^ (64 %), which is similar to the proportion of toxigenic environmental isolates observed in Australia and Europe [85]. A *tcdE* homolog was observed for multiple *tcdB* ^-^ genomes sequenced in this study (Fig. 3); this gene was associated with a holin family protein (WP_021372187) in a phage.

### SNP diversity exists in multiple isolates from the same sample

From six samples (five clinical and one dog), multiple isolates of the same sequence type were sequenced from each to examine the genomic diversity within a single sample. A pairwise comparison of SNPs among isolates with the same sequence type from single samples identified one pairwise SNP difference among genomes isolated from each of two clinical samples (Table S4, zero pairwise SNP differences were identified among genomes with the same sequence type for other samples), suggesting that within-sample diversity exists in some samples and should be considered when identifying transmission events. This result is consistent with another study that identified as many as three SNPs from intra-sample comparative genomic comparisons [86] and with a study identifying an average of 0–2 SNP differences among isolates of the same sequence type originating from dog faecal samples [28].

### ST overlap across all reservoirs

In this study, we identified 17 sequence types that span reservoirs; of these, eight STs were shared across all three reservoirs, whereas nine were shared across just two reservoirs (Fig. 2). These findings may be biassed, however, as sampling was uneven across these three sources. Sequence types spanning all three reservoirs are described below (in order of prevalence, see Fig. 2):

*ST2 is an emerging, diverse, toxigenic ST that showed no signs of transmission*. ST2 isolates have been observed in clinical and environmental samples worldwide [87]. ST2 isolates were collected from human (*n*=42), canine (*n*=7), and environmental (*n*=27) sources in Flagstaff, and ST2 was the most common ST identified in this study. A comparative SNP analysis using an ST2 reference genome (Table S5) indicated that one pair of environmental isolates (ES-S-0022–03, ES-S-0050–04) were separated by zero SNPs; these isolates originated from samples collected 1 week and approximately 4.5 km apart. All other isolates were separated by at least nine SNPs. A screen of the core genome of ST2 (*n*=3478) identified a single CDS (CDR20291_0440) that although not highly conserved in ST2 genomes, was not found in other *C. difficile *.

*ST42 is a globally distributed, toxigenic sequence type found across all reservoirs*. ST42, also known as RT106, DH, and NAP1, is becoming one of the most commonly observed STs of *C. difficile* globally [84]. We sequenced isolates collected from human (*n*=39), canine (*n*=12), and environmental (*n*=23) reservoirs. A comparative SNP analysis using an ST42 reference (Table S5) identified eight clinical isolate pairs that differed by two or fewer SNPs. These are all from a single hospital network and could indicate healthcare-associated transmission although the transmission dynamics are unknown. Genomes from two ST42 environmental isolates (ES-S-0040–03, ES-S-0053–01) were identical at the SNP level; the samples from which these isolates originated were collected approximately 2.25 km and fifteen months apart. There was one clinical/environmental pair (ES-W-0046–03, HS-FS-000334-I-03) that differed by zero SNPs, with samples collected approximately 4 months apart.

The presence of three genomic islands (GIs) unique to ST42/ST28 has been described previously [84]. Our analysis confirmed that CDSs from GI1 are conserved across ST42 genomes from this study and that CDSs from GI2 and GI3 are present but not widely conserved (Table S3). However, a screen of these regions against a diverse set of *C. difficile* genomes demonstrated that CDSs from all three GIs are distributed across other STs, suggesting that they are not unique to ST42/ST28 although the complete GI structure may be.

The population structure of ST42 demonstrates the presence of at least three distinct clades; the largest clade includes the majority of the genomes (*n*=735), including all but one sequenced in this study (clinical isolate HS-FS-000362-I-03) (Fig. S1, available in the online version of this article). The two rarer clades are characterized by a stepwise evolution and CDSs loss, with clade 1b having lost 18 CDSs compared to clade two genomes. Clade two has lost a single gene associated with flavodoxin (WP_021373557). All CDSs differentially present in ST42 genomes are variably conserved across all analysed genomes (Fig. S1 and Table S6), suggesting that they may be present on mobile genetic elements.

*Non-toxigenic ST3 (ST3-NT) isolates are widespread across human, dog, and environmental reservoirs in the Flagstaff area*. The core-genome SNP phylogeny (Fig. 3) demonstrated that ST3 is composed of two separate monophyletic groups. One group of ST3 genomes contains the PaLoc (ST3-T), whereas the other group is non-toxigenic (ST3-NT). ST3-NT genomes were sequenced from clinical (*n*=5), dog (*n*=9), and environmental samples (*n*=14). ST3-NT and ST15 (discussed below) were the most common non-toxigenic clades observed in this study. Comparative SNP analysis with a ST3-NT reference (Table S5) identified a soil isolate (ES-S-0210–01) and a dog isolate (DGF-0227–02) that were identical at the SNP level although they were collected approximately 7 months apart (Table S1). We also identified two ST3-NT dog isolates (DGF-0034–01, DGF-0153–01) that were identical at the SNP level but were collected 9 days and approximately ten kilometres apart. These two isolates differed by only two SNPs from a third dog isolate (DGF-0059–01) that was collected the same day as DGF-0034–01 but at a different location (approximately 2.5 km away from the site of DGF-0034–01). Two patient-derived isolates (HS-FS-000093-I-02, HS-FS-000080-I-02) differed by one SNP, suggesting the transmission of a non-toxigenic genotype; these stool samples were collected on the same day from different patients at the same healthcare facility (Table S1). A screen of the core genome from ST3-NT (*n*=3423), based on an analysis of 144 genomes, failed to identify any core genome CDSs that were specific to the clade.

*ST15 is a non-toxigenic sequence type that has previously been found in dogs*. Genomes were sequenced from ST15 isolates from clinical (*n*=2), dog (*n*=11), and environmental samples (*n*=15). A comparative SNP analysis with a ST15 reference (Table S5) identified environmental isolates that differed by zero (ES-S-0043–03, ES-S-0044–04) and two SNPs (ES-S-0049–04, ES-S-0050–02). In each case the isolates were obtained from samples collected on the same day in close proximity to each other. A dog isolate (DGF-0214–11) and a soil isolate (ES-S-0099–03) that differed by two SNPs were also identified; these isolates were obtained from samples collected almost 4 years apart. A screen of the ST15 core genome (*n*=3472 CDSs), based on 397 total genomes, against other *C. difficile* genomes identified a CbrC family protein (WP_021367084.1) that was unique to ST15; in *E. coli*, overexpression of CbrC results in a Colicin tolerant phenotype [88].

*ST110 is a single locus variant of ST2 primarily found in the environment in Flagstaff*. ST110 isolates were collected from human (*n*=9), canine (*n*=2), and environmental (*n*=12) sources; ST110 differs from ST2 at a single MLST allele (*recA*) and is part of the same lineage on the core genome phylogeny (Fig. 3). A comparative SNP analysis using an ST110 reference genome (Table S5) indicated that all isolates were separated by at least eight SNPs.

*ST8 is a toxigenic ST that was mostly observed in hospital samples in Flagstaff*. ST8 isolates were collected from 20 clinical samples, one canine sample, and one environmental sample. A comparative SNP analysis using an ST8 reference genome (Table S5) identified two sets of clinical isolates that differed by zero SNPs (HS-FS-000387-I-02, HS-FS-000399-I-02 and HS-FS-000405-I-02, HS-FS-000415-I-02). Samples HS-FS-000387 and HS-FS-000399 were collected 1 day apart; samples HS-FS-000405 and HS-FS-000415 were collected within a time period of approximately 3 weeks. Additional pairs of clinical isolates with five or fewer SNP differences were also identified. Among Flagstaff ST8 isolates, at least 12 SNP differences separated isolates from different reservoirs indicating that clinical, environmental and dog isolates were distinct from each other. A screen of the ST8 core genome (*n*=3419 CDSs) failed to identify any CDSs unique to ST8.

*ST26 is a non-toxigenic sequence type*. ST26 genomes were sequenced from clinical (*n*=3), dog (*n*=2), and environmental isolates (*n*=6). A comparative SNP analysis using a relevant reference (Table S5) identified genomes separated by fewer than five SNPs. There were two environmental isolates (ES-S-0121–01, ES-S-0167–01) that both differed by four SNPs from a dog sample isolate (DGF-0201–07); the environmental isolates differed by two SNPs from each other and were collected approximately 2 months apart from different areas around Flagstaff, whereas the dog sample was collected in a veterinary clinic [28]. There was also an environmental/clinical pair (ES-S-0064–01, HS-FS-000346-I-03) that differed by seven SNPs from each other. A screen of toxin genes demonstrated that all ST26 genomes examined in this study were *tcdB* ^-^. A screen of the ST26 core genome (*n*=3693 CDSs), based on 198 genomes, against other *C. difficile* genomes identified an energy-coupling factor transporter gene (WP_021421929.1) that was unique to ST26. Most of the Flagstaff ST26 genomes contained the *tetM* and *ermB* genes associated with antimicrobial resistance (Fig. 3).

*ST236 was rarely observed in Flagstaff*. ST236 is a toxigenic ST. Only five ST236 genomes were identified in this study, although the isolates were obtained from all three reservoirs. A comparative SNP analysis identified two soil isolates (ES-S-0020–03, ES-S-0022–02) that were identical at the SNP level using a ST236 reference (Table S5); these two isolates were taken from soil samples collected ~8.5 km and approximately 2 weeks apart. Each of the two clinical isolates differed by at least 16 SNPs from any other isolate. A screen of the ST236 core genome (*n*=3673 CDSs) against all other *C. difficile* genome assemblies identified a hypothetical protein gene (EGT3900969.1) that was unique to all ST236 genomes.

### ST overlap across two reservoirs

There were nine STs (plus ST3-T) where genomes were sequenced from isolates across two of the three reservoirs (Table S5). These included: ST28, ST6, ST10, ST31, ST35, ST43, ST53, ST54, ST83 and ST3-T (toxigenic ST3, considered separately from ST3-NT here). A comparative SNP analysis on each ST using a relevant reference (Table S5) identified genome pairs separated by five SNPs or fewer for some of these STs: ST3-T, ST6, ST43, ST53. For ST3-T we sequenced 24 clinical isolates and two environmental isolates and identified multiple pairs of isolates separated by two or fewer SNPs. Two pairs of clinical isolates were separated by zero SNPs (HS-FS-000039-I-01, HS-FS-000056-I-01 and HS-FS-000237-I-02, HS-FS-000254-I-02), and five pairs of clinical isolates were separated by two SNP differences. Additionally, hospital isolates (HS-FS-000107-I-05, HS-FS-000237-I-02, HS-FS-000254-I-02, HS-FS-000422-I-02) and one environmental isolate (ES-S-0023–02) differed by two SNPs. We sequenced 21 clinical isolates and one dog isolate for ST6; a comparative SNP analysis indicated that a pair of clinical isolates differed by five SNPs (HS-FS-000296-I-02, HS-FS-000495-I-02). A clinical isolate (HS-FS-000162-I-02) differed by fewer than five SNPs from several other isolates; however, the coverage filter applied by NASP decreased the effective genome search space for confident pair-wise comparisons with this genome. For ST43, we sequenced 16 clinical isolates and one environmental isolate; eight clinical isolates were separated by zero SNPs from at least one other clinical isolate. For ST53, we sequenced 13 clinical isolates and one environmental isolate, and one pair of clinical isolates differed by four SNPs.

### STs in a single reservoir

We identified multiple isolates from a single reservoir for 19 STs: 18 STs with multiple isolates from only clinical samples and one ST with multiple isolates from only environmental samples (Table S5). Isolate pairs differing by two or fewer SNPs were identified for seven of these sequence types isolated from clinical samples. For ‘hypervirulent’ STs that have been associated with severe infections (ST1, ST11, ST67), isolates were only obtained from clinical samples. Additionally, 27 STs were represented by a single clinical isolate; one ST was represented by a single environmental isolate and one ST was represented by a single dog isolate.

### Healthcare-associated transmission identified via genomic-guided epidemiology

We identified 78 clinical genomes (out of a total of 401 clinical genomes) that were in clusters with other clinical genomes at a SNP threshold of 2 or less; using this SNP threshold, ~19 % of isolates were associated with potential healthcare-associated transmission. Isolates from potential hospital acquired infections (HAIs) were associated with ST1 (*n*=28), ST42 (*n*=16), ST43 (*n*=8), ST3-T (*n*=6), ST8 (*n*=4), and ST95 (*n*=4). Other STs contained a single pair of clinical isolates with two or fewer SNPS: ST3-NT, ST34, ST47, ST58, ST67, ST224. Importantly, we only considered SNP thresholds for identifying potential healthcare-associated transmission, we only sampled symptomatic patients and did not examine asymptomatic transmission, and we did not include detailed patient tracing in this analysis.

### Non-toxigenic STs are widespread in Flagstaff samples

In addition to the non-toxigenic STs found across all three reservoirs (ST15, ST26, ST3-NT), we also observed other non-toxigenic STs: ST109 (two clinical isolates), ST205 (three environmental isolates), ST31 (one dog, one environmental isolate), ST83 (one hospital, one environmental isolate), ST100 (one environmental isolate), ST29 (one dog isolate), and ST867 (one clinical isolate). A *tcdB* screen against genomes in GenBank confirmed that public genomes sequenced from these STs are also missing the *tcdB* gene except for ST867 for which no public genomes were available. In total, 79 non-toxigenic isolates (~14 %) were identified as part of this study.

## Discussion

Although once thought of mostly as a hospital-acquired pathogen [89], growing evidence supports the role of community acquisition in *C. difficile* infection [90]. Most *C. difficile* sequencing and surveillance has focused on clinical isolates [13, 35], although molecular surveys have identified *C. difficile* DNA in soil and water [35], canines [28], raw meat [91], the soles of shoes [92], and farm animals [43]. The routes of transmission between reservoirs, especially as they relate to human CDI, are not entirely clear and cannot be completely resolved with whole genome sequencing alone. In this study, we isolated toxigenic and non-toxigenic *C. difficile* across clinical, environmental, and canine reservoirs in Flagstaff, AZ, USA to understand the distribution of *C. difficile* and provide insights into potential transmission events. Canines were chosen as a non-human animal reservoir due to their close interaction with humans, although additional *C. difficile* diversity likely exists in other animal reservoirs, including felines [93]. Flagstaff represents an ideal study site for these types of studies [94] as it is geographically isolated and has a single hospital network that partnered with us to transfer *tcdB* ^+^ positive stool samples.

We used high-resolution genomic epidemiology to identify potential clinical transmission of *C. difficile *. The overall proportion of clinical isolates associated with potential healthcare associated infections in this study was ~19 %, although we only sampled symptomatic cases and did not capture asymptomatic carriage and transmission. Also, we based this estimate solely on genomic comparisons and did not include other epidemiological information. Potential clinical transmission of *C. difficile* was identified within 11 STs, including the putative transmission of non-toxigenic, ST3-NT *C. difficile* isolates. Our sampling strategy involved the isolation and independent processing of ten colonies per specimen, which allowed us to identify toxigenic and non-toxigenic *C. difficile* mixtures (e.g. ST1 and ST26) in a single clinical sample; mixture of toxigenic and non-toxigenic *C. difficile* in a single sample has been observed previously [95]. We were also able identify pairwise SNP differences among isolates within the same sequence type from the same clinical samples. SNP differences among intra-sample isolates of the same sequence type have been previously reported [28, 86]. Consideration of the diversity of *C. difficile* isolates within a single clinical sample has important implications when attempting to identify transmission events. Selecting a single isolate from a sample may miss strains involved in transmission events and relying on sub-genomic genotyping methods (e.g. MLST) could underestimate diversity between isolates and overestimate potential transmission events.

A deep sampling of clinical, environmental and dog sources allowed us to identify many STs that spanned multiple reservoirs; the mechanisms behind spread between reservoirs was not investigated in this study but could be facilitated by the close interaction between humans and companion animals, both of which interact with soil in the environment. We identified few potential transmission events between reservoirs (Table S5). We only identified two environmental isolates (one ST42 and one ST3-T isolate) with two or fewer SNP differences to a clinical isolate. The potential for transmission between dogs and humans has been suggested [96], and one study did identify transmission between an infant and a dog with diarrhoea in the same home [44]. In our study, we did not identify closely related isolates from dogs and clinical samples. However, we did not sample the social networks of CDI patients, which may have identified transmission events. In two instances (ST3-NT and ST15) we observed environmental and dog isolates differing by two or fewer SNPs. A study in China identified close genetic overlap with *C. difficile* isolates across all three reservoirs [42], although they used a core genome analysis with a distant outgroup and the effective core genome space was likely too small to clearly identify transmissions (see below). Although one study sampled the environment, pigs, and farmers within a single farm and failed to identify any zoonotic transmission [97], another study found genotypic overlap between isolates from humans and non-human animals [43]. It is unclear if zoonotic events are rare in *C. difficile *, the events involve asymptomatic infection, and/or if under sampling is simply missing these events. Future work using more comprehensive sampling, such as sampling both symptomatic and asymptomatic individuals within a social network, companion animals of multiple species, domesticated animals, food sources and the environment, may better elucidate the frequency of cross reservoir transmission.

Non-toxigenic *C. difficile* (NTCD) were commonly identified in this study. NTCD were previously detected in dog faecal samples in Flagstaff [48] and were identified in all three reservoirs in this study. We identified NTCD in two clinical stool samples that were *tcdB* ^+^ based on clinical diagnostics. NTCD have been shown to effectively colonize the mammalian gut [98], suggesting that the absence of the toxins does not inhibit the colonization and spread of the species, and colonized NTCD may actually have a fitness advantage over toxigenic *C. difficile* [99]. The presence of NTCD has been previously documented in healthy subjects [100] and could potentially provide a protective effect [101] through antagonism [98]. Challenge studies of hamsters colonized with NTCD demonstrated strong protection against disease with toxigenic strains [102], demonstrating the potential of NTCD to serve as a biotherapeutic [103]. The presence of toxin gene homologs (e.g*. tcdA*, *tcdB*) in closely related and ancestral *Clostridioides* species [55, 68] suggests that toxin genes have been lost multiple, independent times in the evolution of *C. difficile *.

Genotyping of clinical *C. difficile* has largely focused on ribotyping, a fingerprinting tool that can be used for strain matching [45], but is not backward compatible with whole genome sequence data [58, 104]; as such it was not used in this study. Sequence typing based on a published MLST scheme [49] is compatible with whole genome sequencing data and has also been effectively used to group strains and provide low resolution genomic epidemiology information [105]. However, MLST based phylogenies have been shown to poorly reflect the core genome phylogeny [106] and phenotypic diversity can exist within a single *C. difficile* ST [65], suggesting that MLST data alone are insufficient to understand the diversity within a population. In this study, we confirmed that ST3 is polyphyletic (Fig. 3), which would confuse MLST-based phylogenies. Additionally, some STs have been shown to contain multiple ribotypes [50] and some ribotypes are polyphyletic [107]. With the widespread application of whole genome sequencing, typing based methods, such as rMLST [108], cgMLST [51], and cgSNPs, should be used to maximize the potential to classify strains and identify transmission events. The challenge is linking current results to previous data that typed strains based on ribotype or MLST alone.

As in this study, the transmission of *C. difficile* between humans or between reservoirs has been determined using comparative SNP thresholds [109]. Shared cgMLST alleles have also been used to investigate transmission [51, 96]; a comparative analysis suggests that similar results were obtained across the two methods [110]. Eyre *et al*. suggested that a pairwise SNP threshold of ≤2 indicates a transmission event, whereas a SNP threshold of >10 indicates more genetically distinct strains [13]. Others have confirmed that a pairwise SNP threshold from 0 to 10 indicates closely related strains [111]. Although using SNP thresholds in a spore forming bacterium without a strict molecular clock is challenging [112], a SNP threshold of <10 seems appropriate for at least identifying strains with a similar genomic background. In this study, we identified genomes separated by ≤2 SNPs that were collected months apart; this demonstrates the difficulty in the identification of transmission in the absence of orthogonal, supporting information. From a methodological perspective, determining pairwise SNPs should always be provided in the context of the genomic search space from which SNPs were called. SNPs are often called from the core genome, which is the conserved gene content of all analysed genomes [113] and can be diminished by the inclusion of poor genome assemblies; this likely explains the widely variable core genome sizes reported for *C. difficile* [52, 114]. In this study, we provide the size of the genomic search space for SNP comparisons for each ST (Table S5), as determined by NASP [70], to put these results in context as well as for comparison to other studies. NASP allows for a comprehensive, reference-based, pairwise SNP comparison between all sequenced genomes, maximizing the search space for comparative SNP analyses. We also used a relevant reference genome for each ST comparison, as the choice of reference genome can drastically affect not only the size of the genomic search space, but also how many sequence reads can be mapped and therefore used for SNP calling [70, 115].

This study provides a One Health framework for comprehensive surveillance of *C. difficile* in a single community, as well as for robust methods for detecting potential transmission events. Although overlap of closely related *C. difficile* isolates across reservoirs was detected in a few instances using comparative genomic methods, the directionality of transmission could not be determined. Future work could include more intensive, longitudinal sampling of individuals and additional sources, such as food and domesticated animals. At the minimum, our results demonstrate that many of the most commonly observed STs can inhabit all sampled reservoirs. These results can inform mitigation efforts focused on preventing hospital acquisition, or for informing the public on good hygiene when disposing of pet waste. Additional functional work directed at the efficiency of colonization and toxin production will further explore differences in similar isolates obtained from different reservoirs.

## Supplementary Data

Supplementary material 1

Supplementary material 2

## References

[1] George WL, Sutter VL, Goldstein EJ, Ludwig SL, Finegold SM (1978). Aetiology of antimicrobial-agent-associated colitis. Lancet.

[2] George RH, Symonds JM, Dimock F, Brown JD, Arabi Y (1978). Identification of *Clostridium difficile* as a cause of pseudomembranous colitis. Br Med J.

[3] Larson HE, Price AB, Honour P, Borriello SP (1978). *Clostridium difficile* and the aetiology of pseudomembranous colitis. Lancet.

[4] Bartlett JG, Moon N, Chang TW, Taylor N, Onderdonk AB (1978). Role of *Clostridium difficile* in antibiotic-associated pseudomembranous colitis. Gastroenterology.

[5] Hammond GA, Johnson JL (1995). The toxigenic element of *Clostridium difficile* strain VPI 10463. Microb Pathog.

[6] Dingle KE, Elliott B, Robinson E, Griffiths D, Eyre DW (2014). Evolutionary history of the *Clostridium difficile* pathogenicity locus. Genome Biol Evol.

[7] Mani N, Dupuy B (2001). Regulation of toxin synthesis in *Clostridium difficile* by an alternative RNA polymerase sigma factor. Proc Natl Acad Sci.

[8] Matamouros S, England P, Dupuy B (2007). *Clostridium difficile* toxin expression is inhibited by the novel regulator TcdC. Mol Microbiol.

[9] Tan KS, Wee BY, Song KP (2001). Evidence for holin function of tcdE gene in the pathogenicity of *Clostridium difficile*. J Med Microbiol.

[10] Gerding DN, Johnson S, Rupnik M, Aktories K (2014). *Clostridium difficile* binary toxin CDT: mechanism, epidemiology, and potential clinical importance. Gut Microbes.

[11] Brazier JS (1998). The epidemiology and typing of *Clostridium difficile*. J Antimicrob Chemother.

[12] Kuijper EJ, Coignard B, Tüll P (2006). Emergence of *Clostridium difficile*-associated disease in North America and Europe. Clin Microbiol Infect.

[13] Eyre DW, Cule ML, Wilson DJ, Griffiths D, Vaughan A (2013). Diverse sources of *C. difficile* infection identified on whole-genome sequencing. N Engl J Med.

[14] Dingle KE, Didelot X, Quan TP, Eyre DW, Stoesser N (2017). Effects of control interventions on *Clostridium difficile* infection in England: an observational study. Lancet Infect Dis.

[15] Feazel LM, Malhotra A, Perencevich EN, Kaboli P, Diekema DJ (2014). Effect of antibiotic stewardship programmes on *Clostridium difficile* incidence: a systematic review and meta-analysis. J Antimicrob Chemother.

[16] Smits WK, Lyras D, Lacy DB, Wilcox MH, Kuijper EJ (2016). *Clostridium difficile* infection. Nat Rev Dis Primers.

[17] Lim SC, Knight DR, Riley TV (2020). *Clostridium difficile* and one health. Clin Microbiol Infect.

[18] Ofori E, Ramai D, Dhawan M, Mustafa F, Gasperino J (2018). Community-acquired *Clostridium difficile*: epidemiology, ribotype, risk factors, hospital and intensive care unit outcomes, and current and emerging therapies. J Hosp Infect.

[19] Khanna S, Pardi DS, Aronson SL, Kammer PP, Orenstein R (2012). The epidemiology of community-acquired *Clostridium difficile* infection: a population-based study. Am J Gastroenterol.

[20] Hensgens MPM, Keessen EC, Squire MM, Riley TV, Koene MGJ (2012). Clostridium difficile infection in the community: a zoonotic disease?. Clin Microbiol Infect.

[21] Rodriguez-Palacios A, Stämpfli HR, Duffield T, Peregrine AS, Trotz-Williams LA (2006). *Clostridium difficile* PCR ribotypes in calves, Canada. Emerg Infect Dis.

[22] Knight DR, Thean S, Putsathit P, Fenwick S, Riley TV (2013). Cross-sectional study reveals high prevalence of *Clostridium difficile* non-PCR ribotype 078 strains in Australian veal calves at slaughter. Appl Environ Microbiol.

[23] Rodriguez C, Hakimi D-E, Vanleyssem R, Taminiau B, Van Broeck J (2017). *Clostridium difficile* in beef cattle farms, farmers and their environment: assessing the spread of the bacterium. Vet Microbiol.

[24] Hain-Saunders NMR, Knight DR, Bruce M, Riley TV (2022). *Clostridioides difficile* infection and one health: an equine perspective. Environ Microbiol.

[25] Knight DR, Squire MM, Riley TV (2015). Nationwide surveillance study of *Clostridium difficile* in Australian neonatal pigs shows high prevalence and heterogeneity of PCR ribotypes. Appl Environ Microbiol.

[26] Keel K, Brazier JS, Post KW, Weese S, Songer JG (2007). Prevalence of PCR ribotypes among *Clostridium difficile* isolates from pigs, calves, and other species. J Clin Microbiol.

[27] Zidaric V, Zemljic M, Janezic S, Kocuvan A, Rupnik M (2008). High diversity of *Clostridium difficile* genotypes isolated from a single poultry farm producing replacement laying hens. Anaerobe.

[28] Stone NE, Sidak-Loftis LC, Sahl JW, Vazquez AJ, Wiggins KB (2016). More than 50% of *Clostridium difficile* isolates from pet dogs in Flagstaff, USA, carry toxigenic genotypes. PLoS One.

[29] Rabold D, Espelage W, Abu Sin M, Eckmanns T, Schneeberg A (2018). The zoonotic potential of *Clostridium difficile* from small companion animals and their owners. PLoS One.

[30] Rodriguez C, Taminiau B, Bouchafa L, Romijn S, Van Broeck J (2019). *Clostridium difficile* beyond stools: dog nasal discharge as a possible new vector of bacterial transmission. Heliyon.

[31] Orden C, Neila C, Blanco JL, Álvarez-Pérez S, Harmanus C (2018). Recreational sandboxes for children and dogs can be a source of epidemic ribotypes of clostridium difficile. Zoonoses Public Health.

[32] Shivaperumal N, Chang BJ, Riley TV (2020). High prevalence of clostridium difficile in home gardens in western Australia. Appl Environ Microbiol.

[33] Zidaric V, Beigot S, Lapajne S, Rupnik M (2010). The occurrence and high diversity of clostridium difficile genotypes in rivers. Anaerobe.

[34] Janezic S, Potocnik M, Zidaric V, Rupnik M (2016). Highly divergent clostridium difficile strains isolated from the environment. PLoS One.

[35] Janezic S, Smrke J, Rupnik M (2020). Isolation of *Clostridioides difficile* from different outdoor sites in the domestic environment. Anaerobe.

[36] Lim S-C, Hain-Saunders NMR, Imwattana K, Putsathit P, Collins DA (2022). Genetically related *Clostridium difficile* from water sources and human CDI cases revealed by whole-genome sequencing. Environ Microbiol.

[37] Rodriguez-Palacios A, Lejeune JT (2011). Moist-heat resistance, spore aging, and superdormancy in *Clostridium difficile*. Appl Environ Microbiol.

[38] Knight DR, Kullin B, Androga GO, Barbut F, Eckert C (2019). Evolutionary and genomic insights into *Clostridioides difficile* sequence type 11: a diverse zoonotic and antimicrobial-resistant lineage of global one health importance. mBio.

[39] Knight DR, Riley TV (2019). Genomic delineation of zoonotic origins of. Front Public Health.

[40] Knetsch CW, Kumar N, Forster SC, Connor TR, Browne HP (2018). Zoonotic transfer of clostridium difficile harboring antimicrobial resistance between farm animals and humans. J Clin Microbiol.

[41] Turner NA, Smith BA, Lewis SS (2019). Novel and emerging sources of *Clostridioides difficile* infection. PLoS Pathog.

[42] Zhou Y, Zhou W, Xiao T, Chen Y, Lv T (2021). Comparative genomic and transmission analysis of *Clostridioides difficile* between environmental, animal, and clinical sources in China. Emerg Microbes Infect.

[43] Knetsch CW, Connor TR, Mutreja A, van Dorp SM, Sanders IM (2014). Whole genome sequencing reveals potential spread of clostridium difficile between humans and farm animals in the Netherlands, 2002 to 2011. Euro Surveill.

[44] Rodríguez-Pallares S, Fernández-Palacios P, Jurado-Tarifa E, Arroyo F, Rodríguez-Iglesias MA (2022). Transmission of toxigenic Clostridiodes difficile between a pet dog with diarrhea and a 10-month-old infant. Anaerobe.

[45] Bidet P, Barbut F, Lalande V, Burghoffer B, Petit JC (1999). Development of a new PCR-ribotyping method for clostridium difficile based on ribosomal RNA gene sequencing. FEMS Microbiol Lett.

[46] Knetsch CW, Lawley TD, Hensgens MP, Corver J, Wilcox MW (2013). Current application and future perspectives of molecular typing methods to study clostridium difficile infections. Euro Surveill.

[47] Stabler RA, He M, Dawson L, Martin M, Valiente E (2009). Comparative genome and phenotypic analysis of clostridium difficile 027 strains provides insight into the evolution of a hypervirulent bacterium. Genome Biol.

[48] Dingle KE, Griffiths D, Didelot X, Evans J, Vaughan A (2011). Clinical clostridium difficile: clonality and pathogenicity locus diversity. PLoS One.

[49] Griffiths D, Fawley W, Kachrimanidou M, Bowden R, Crook DW (2010). Multilocus sequence typing of *Clostridium difficile*. J Clin Microbiol.

[50] Janezic S, Rupnik M (2015). Genomic diversity of *Clostridium difficile* strains. Res Microbiol.

[51] Bletz S, Janezic S, Harmsen D, Rupnik M, Mellmann A (2018). Defining and evaluating a core genome multilocus sequence typing scheme for genome-wide typing of *Clostridium difficile*. J Clin Microbiol.

[52] Scaria J, Ponnala L, Janvilisri T, Yan W, Mueller LA (2010). Analysis of ultra low genome conservation in *Clostridium difficile*. PLoS One.

[53] O’Grady K, Knight DR, Riley TV (2021). Antimicrobial resistance in *Clostridioides difficile*. Eur J Clin Microbiol Infect Dis.

[54] Lewis BB, Carter RA, Ling L, Leiner I, Taur Y (2017). Pathogenicity locus, core genome, and accessory Gene contributions to virulence. MBio.

[55] Williamson CHD, Stone NE, Nunnally AE, Roe CC, Vazquez AJ (2022). Identification of novel, cryptic *Clostridioides* species isolates from environmental samples collected from diverse geographical locations. Microb Genom.

[56] Pritchard L, Glover RH, Humphris S, Elphinstone JG, Toth IK (2016). Genomics and taxonomy in diagnostics for food security: soft-rotting enterobacterial plant pathogens. Anal Methods.

[57] Jain C, Rodriguez-R LM, Phillippy AM, Konstantinidis KT, Aluru S (2018). High throughput ANI analysis of 90K prokaryotic genomes reveals clear species boundaries. Nat Commun.

[58] Williamson CHD, Stone NE, Nunnally AE, Hornstra HM, Wagner DM (2019). A global to local genomics analysis of *Clostridioides difficile* ST1/RT027 identifies cryptic transmission events in a northern Arizona healthcare network. Microb Genom.

[59] Bankevich A, Nurk S, Antipov D, Gurevich AA, Dvorkin M (2012). SPAdes: a new genome assembly algorithm and its applications to single-cell sequencing. J Comput Biol.

[60] Walker BJ, Abeel T, Shea T, Priest M, Abouelliel A (2014). Pilon: an integrated tool for comprehensive microbial variant detection and genome assembly improvement. PLoS One.

[61] Li H (2009). Minimap2: pairwise alignment for nucleotide sequences. Bioinformatics.

[62] Li H, Handsaker B, Wysoker A, Fennell T, Ruan J (2009). The sequence alignment/Map format and SAMtools. Bioinformatics.

[63] Sayers EW, Agarwala R, Bolton EE, Brister JR, Canese K (2019). Database resources of the national center for biotechnology information. Nucleic Acids Res.

[64] Camacho C, Coulouris G, Avagyan V, Ma N, Papadopoulos J (2009). BLAST+: architecture and applications. BMC Bioinformatics.

[65] Stone NE, Nunnally AE, Jimenez V, Cope EK, Sahl JW (2019). Domestic canines do not display evidence of gut microbial dysbiosis in the presence of *Clostridioides* (Clostridium) difficile, despite cellular susceptibility to its toxins. Anaerobe.

[66] Souvorov A, Agarwala R, Lipman DJ (2018). SKESA: strategic k-mer extension for scrupulous assemblies. Genome Biol.

[67] Kurtz S, Phillippy A, Delcher AL, Smoot M, Shumway M (2004). Versatile and open software for comparing large genomes. Genome Biol.

[68] Knight DR, Imwattana K, Kullin B, Guerrero-Araya E, Paredes-Sabja D (2021). Major genetic discontinuity and novel toxigenic species in *Clostridioides difficile* taxonomy. Elife.

[69] Guerrero-Araya E, Muñoz M, Rodríguez C, Paredes-Sabja D (2021). FastMLST: a multi-core tool for multilocus sequence typing of draft genome assemblies. Bioinform Biol Insights.

[70] Sahl JW, Lemmer D, Travis J, Schupp JM, Gillece JD (2016). NASP: an accurate, rapid method for the identification of SNPs in WGS datasets that supports flexible input and output formats. Microb Genom.

[71] Minh BQ, Schmidt HA, Chernomor O, Schrempf D, Woodhams MD (2020). Corrigendum to: IQ-TREE 2: new models and efficient methods for phylogenetic inference in the genomic era. Mol Biol Evol.

[72] Kalyaanamoorthy S, Minh BQ, Wong TKF, von Haeseler A, Jermiin LS (2017). ModelFinder: fast model selection for accurate phylogenetic estimates. Nat Methods.

[73] Letunic I, Bork P (2016). Interactive tree of life (iTOL) v3: an online tool for the display and annotation of phylogenetic and other trees. Nucleic Acids Res.

[74] McKenna A, Hanna M, Banks E, Sivachenko A, Cibulskis K (2010). The genome analysis toolkit: a MapReduce framework for analyzing next-generation DNA sequencing data. Genome Res.

[75] Schwengers O, Jelonek L, Dieckmann MA, Beyvers S, Blom J (2021). Bakta: rapid and standardized annotation of bacterial genomes via alignment-free sequence identification. Microb Genom.

[76] Tonkin-Hill G, MacAlasdair N, Ruis C, Weimann A, Horesh G (2020). Producing polished prokaryotic pangenomes with the Panaroo pipeline. Genome Biol.

[77] Sahl JW, Caporaso JG, Rasko DA, Keim P (2014). The large-scale blast score ratio (LS-BSR) pipeline: a method to rapidly compare genetic content between bacterial genomes. PeerJ.

[78] Kent WJ (2002). BLAT--the BLAST-like alignment tool. Genome Res.

[79] Rasko DA, Myers GSA, Ravel J (2005). Visualization of comparative genomic analyses by BLAST score ratio. BMC Bioinformatics.

[80] Roberts MC (1996). Tetracycline resistance determinants: mechanisms of action, regulation of expression, genetic mobility, and distribution. FEMS Microbiol Rev.

[81] Banawas SS (2018). Infections: a global overview of drug sensitivity and resistance mechanisms. Biomed Res Int.

[82] Imwattana K, Putsathit P, Knight DR, Kiratisin P, Riley TV (2021). Molecular characterization of, and antimicrobial resistance in, *Clostridioides difficile* from Thailand, 2017-2018. Microb Drug Resist.

[83] Cizek A, Masarikova M, Mares J, Brajerova M, Krutova M (2022). Detection of plasmid-mediated resistance to metronidazole in *Clostridioides difficile* from river water. Microbiol Spectr.

[84] Roxas BAP, Roxas JL, Claus-Walker R, Harishankar A, Mansoor A (2020). Phylogenomic analysis of *Clostridioides difficile* ribotype 106 strains reveals novel genetic islands and emergent phenotypes. Sci Rep.

[85] Lim S-C, Collins DA, Imwattana K, Knight DR, Perumalsamy S (2022). Whole-genome sequencing links clostridium (*Clostridioides*) difficile in a single hospital to diverse environmental sources in the community. J Appl Microbiol.

[86] Balaji A, Ozer EA, Kociolek LK (2019). *Clostridioides difficile* whole-genome sequencing reveals limited within-host genetic diversity in a pediatric cohort. J Clin Microbiol.

[87] Miles-Jay A, Young VB, Pamer EG, Savidge TC, Kamboj M (2021). A multisite genomic epidemiology study of *Clostridioides difficile* infections in the USA supports differential roles of healthcare versus community spread for two common strains. Microb Genom.

[88] Cariss SJL, Constantinidou C, Patel MD, Takebayashi Y, Hobman JL (2010). YieJ (CbrC) mediates CreBC-dependent colicin E2 tolerance in *Escherichia coli*. J Bacteriol.

[89] Jobe BA, Grasley A, Deveney KE, Deveney CW, Sheppard BC (1995). Clostridium difficile colitis: an increasing hospital-acquired illness. Am J Surg.

[90] Gupta A, Khanna S (2014). Community-acquired clostridium difficile infection: an increasing public health threat. Infect Drug Resist.

[91] Candel-Pérez C, Ros-Berruezo G, Martínez-Graciá C (2019). A review of *Clostridioides* [Clostridium] difficile occurrence through the food chain. Food Microbiol.

[92] Janezic S, Mlakar S, Rupnik M (2018). Dissemination of clostridium difficile spores between environment and households: dog paws and shoes. Zoonoses Public Health.

[93] Clooten J, Kruth S, Arroyo L, Weese JS (2008). Prevalence and risk factors for clostridium difficile colonization in dogs and cats hospitalized in an intensive care unit. Vet Microbiol.

[94] Davis GS, Waits K, Nordstrom L, Weaver B, Aziz M (2015). Intermingled *Klebsiella pneumoniae* populations between retail meats and human urinary tract infections. Clin Infect Dis.

[95] Behroozian AA, Chludzinski JP, Lo ES, Ewing SA, Waslawski S (2013). Detection of mixed populations of clostridium difficile from symptomatic patients using capillary-based polymerase chain reaction ribotyping. Infect Control Hosp Epidemiol.

[96] Bjöersdorff OG, Lindberg S, Kiil K, Persson S, Guardabassi L (2021). Dogs are carriers of *Clostridioides difficile* lineages associated with human community-acquired infections. Anaerobe.

[97] Alves F, Nunes A, Castro R, Sequeira A, Moreira O (2022). Assessment of the transmission dynamics of *Clostridioides difficile* in a farm environment reveals the presence of a new toxigenic strain connected to swine production. Front Microbiol.

[98] Wilson KH, Sheagren JN (1983). Antagonism of toxigenic clostridium difficile by nontoxigenic *C. difficile*. J Infect Dis.

[99] Borriello SP, Barclay FE (1985). Protection of hamsters against clostridium difficile ileocaecitis by prior colonisation with non-pathogenic strains. J Med Microbiol.

[100] Natarajan M, Walk ST, Young VB, Aronoff DM (2013). A clinical and epidemiological review of non-toxigenic *Clostridium difficile*. Anaerobe.

[101] Villano SA, Seiberling M, Tatarowicz W, Monnot-Chase E, Gerding DN (2012). Evaluation of an oral suspension of VP20621, spores of nontoxigenic *Clostridium difficile* strain M3, in healthy subjects. Antimicrob Agents Chemother.

[102] Nagaro KJ, Phillips ST, Cheknis AK, Sambol SP, Zukowski WE (2013). Nontoxigenic *Clostridium difficile* protects hamsters against challenge with historic and epidemic strains of toxigenic BI/NAP1/027 C. difficile. Antimicrob Agents Chemother.

[103] Gerding DN, Sambol SP, Johnson S (2018). Non-toxigenic *Clostridioides* (Formerly Clostridium) difficile for prevention of *C. difficile* infection: from bench to bedside back to bench and back to bedside. Front Microbiol.

[104] Seth-Smith HMB, Biggel M, Roloff T, Hinic V, Bodmer T (2021). Transition from PCR-ribotyping to whole genome sequencing based typing of *Clostridioides difficile*. Front Cell Infect Microbiol.

[105] Ngamskulrungroj P, Sanmee S, Putsathit P, Piewngam P, Elliott B (2015). Correction: molecular epidemiology of *Clostridium difficile* infection in a large teaching hospital in Thailand. PLoS One.

[106] Sahl JW, Matalka MN, Rasko DA (2012). Phylomark, a tool to identify conserved phylogenetic markers from whole-genome alignments. Appl Environ Microbiol.

[107] Ducarmon QR, van der Bruggen T, Harmanus C, Sanders IMJG, Daenen LGM (2022). *Clostridioides difficile* PCR ribotype 151 is polyphyletic and includes pathogenic isolates from cryptic clade C-II with mono-toxin pathogenicity loci that can escape routine diagnostics. bioRxiv.

[108] Jolley KA, Bliss CM, Bennett JS, Bratcher HB, Brehony C (2012). Ribosomal multilocus sequence typing: universal characterization of bacteria from domain to strain. Microbiology.

[109] García-Fernández S, Frentrup M, Steglich M, Gonzaga A, Cobo M (2019). Whole-genome sequencing reveals nosocomial *Clostridioides difficile* transmission and a previously unsuspected epidemic scenario. Sci Rep.

[110] Frentrup M, Zhou Z, Steglich M, Meier-Kolthoff JP, Göker M (2020). A publicly accessible database for *Clostridioides difficile* genome sequences supports tracing of transmission chains and epidemics. Microb Genom.

[111] Mawer DPC, Eyre DW, Griffiths D, Fawley WN, Martin JSH (2017). Contribution to clostridium difficile transmission of symptomatic patients with toxigenic strains who are fecal toxin negative. Clin Infect Dis.

[112] Weller C, Wu M (2015). A generation-time effect on the rate of molecular evolution in bacteria. Evolution.

[113] Callister SJ, McCue LA, Turse JE, Monroe ME, Auberry KJ (2008). Comparative bacterial proteomics: analysis of the core genome concept. PLoS One.

[114] Forgetta V, Oughton MT, Marquis P, Brukner I, Blanchette R (2011). Fourteen-genome comparison identifies DNA markers for severe-disease-associated strains of clostridium difficile. J Clin Microbiol.

[115] Bertels F, Silander OK, Pachkov M, Rainey PB, van Nimwegen E (2014). Automated reconstruction of whole-genome phylogenies from short-sequence reads. Mol Biol Evol.

