# Supplementary material for: A local-scale One Health genomic surveillance of Clostridioides difficile demonstrates highly related strains from humans, canines, and the environment
Source: Microb Genom. 2023 Jun 22;9(6):mgen001046. doi: 10.1099/mgen.0.001046 (PMC10327504; doi:10.1099/mgen.0.001046)
Supplement: Supplementary material 1 [file mgen-9-1046-s001.pdf]

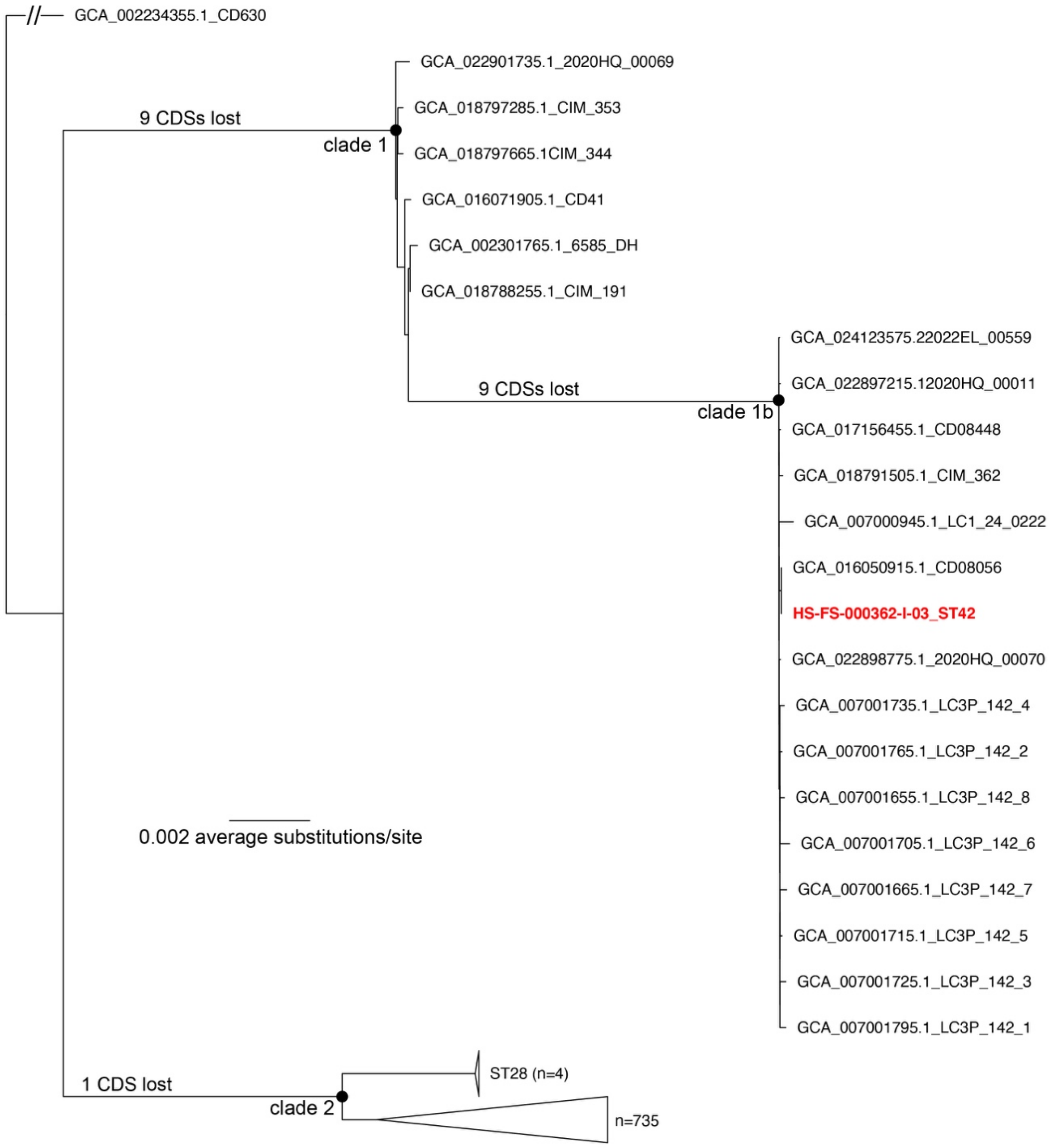

Figure S1. Core genome SNP phylogeny of ST42 and ST28 genomes. GCA_002235.1_CD630 (ST54) is shown as an outgroup. ST28 genomes (n=4) are present in a single clade; all other genomes are ST42. Clades are labeled 1, 1b and 2; loss of coding regions within clades (based upon LS-BSR analyses – see main text) is displayed along branches.
